# Physicochemical controls on the initiation of phytoplankton bloom during the winter monsoon in the Arabian Sea

**DOI:** 10.1038/s41598-021-92897-3

**Published:** 2021-06-29

**Authors:** R. S. Lakshmi, Satya Prakash, Aneesh A. Lotliker, Sanjiba K. Baliarsingh, Alakes Samanta, Teesha Mathew, Abhisek Chatterjee, Biraja K. Sahu, T. M. Balakrishnan Nair

**Affiliations:** 1 Indian National Centre for Ocean Information Services, Ministry of Earth Sciences, Government of India, Hyderbad, 500090 India; 2 Kerala University of Fisheries and Ocean Studies (KUFOS), Kochi, 682506 India; 3 Atal Centre for Ocean Science and Technology for Islands, National Institute of Ocean Technology, Ministry of Earth Sciences, Government of India, Port Blair, Andaman and Nicobar Islands 744103 India

**Keywords:** Ocean sciences, Marine biology, Marine chemistry, Physical oceanography

## Abstract

Occurrence of phytoplankton bloom in the northern Arabian Sea (NAS) during the winter monsoon is perplexing. The convective mixing leads to a deeper and well-oxygenated (> 95% saturation) mixed layer. We encountered low chlorophyll conditions though the nutrient conditions were favorable for a bloom. The mean ratio of silicate (Si) to DIN (Dissolved Inorganic Nitrogen: nitrate + nitrite + ammonium) in the euphotic zone was 0.52 indicating a “silicate-stressed” condition for the proliferation of diatoms. Also, the euphotic depth was much shallower (~ 49 m) than the mixed layer (~ 110 m) suggesting the Sverdrup critical depth limitation in the NAS. We show that the bloom in this region initiates only when the mixed layer shoals towards the euphotic zone. Our observations further suggest that two primary factors, the stoichiometric ratio of nutrients, especially the Si/DIN ratio, in the mixed layer and re-stratification of the upper water column, govern the phytoplankton blooming in NAS during the later winter monsoon. The important finding of the present study is that the Sverdrup’s critical depth limitation gives rise to the observed low chl-*a* concentration in the NAS, despite having enough nutrients.

## Introduction

The Arabian Sea is one of the most productive basins of the world ocean. The productivity regime of the Arabian Sea is predominantly governed by seasonally reversing monsoonal winds and associated physical modulation in the water column^1^. The northern sector of this basin is more productive during the winter monsoon and experiences recurrent high-biomass phytoplankton blooms^2–4^. In general, north-easterly continental cold-dry wind enhances evaporation and consequent densification of the surface waters resulting in strong convective mixing^5,6^. The deeper mixed layer (~ 125 m; Dickey et al.^7^ and Wiggert et al.^8^), often deeper than the seasonal nitracline, leads to entrainment of nutrients into the homogeneous upper layer^1,5,8,9^. The primary production in the Arabian Sea is mainly driven by the availability of nutrients, and the absence of which can cause a decrease in the phytoplankton biomass^5,10^. Among all the nutrients, nitrate is the major nutrient that regulates the primary production in the northeastern Arabian Sea^10,11^. Moreover, this basin is known to be a strong denitrification zone which causes an intense loss of fixed nitrogen from the system^12^. Morrison et al.^11^ has reported that fixed nitrogen is more limiting compared to phosphate. By the end of the winter monsoon, silicate input to the surface decreases because of the re-stratification of water column^13^ which causes a community shift of phytoplankton biomass^14–18^.

Diatom is one of the dominant phytoplankton groups believed to contribute to winter blooms in the northern Arabian Sea^19^. However, since the early 2000s, a mixotrophic dinoflagellate, green *Noctiluca Scnitillans* (hereafter *Noctiluca*) emerged as a predominant contributor to the mixed algal bloom episodes in the northern Arabian Sea^20^. Several studies have reported occurrences of *Noctiluca* bloom in the northern Arabian Sea^3,16–18,21–24^. Gomes et al.^16,18^ suggested that influx of low oxygen water into the surface layer has fuelled the rapid increase of *Noctiluca* in the northern Arabian Sea during the winter monsoon. Prakash et al.^13^, Lotliker et al.^3^, and Sarma et al.^25^, however, argued that skewed silicate to nitrate ratio in the region, primarily controlled by the varying intensity of winter convective mixing, provides a niche for *Noctiluca* to dominate over diatoms. The *Noctiluca* blooms also have been reported to be associated with dissolved oxygen depletion and fish mortality^2,26^. The absence of diatoms and *Noctiluca* in the water column can severely impact the total primary production in the northern Arabian Sea during the winter monsoon^19,22,27^.

Another important and inevitable factor other than nutrients that limits the primary production is the availability of light. Sverdrup^28^ found that the phytoplankton blooming is limited by light, where strong turbulence exists. The critical depth criteria formulated by him analyse the relationship between irradiance and mixed layer depth in determining productivity. Deeper mixed layer induced by strong mixing causes the phytoplankton to utilise a limited amount of light and hence decreases productivity. Many past studies have considered this hypothesis to study the bloom dynamics throughout the world ocean^29–31^.

**Figure 1:**
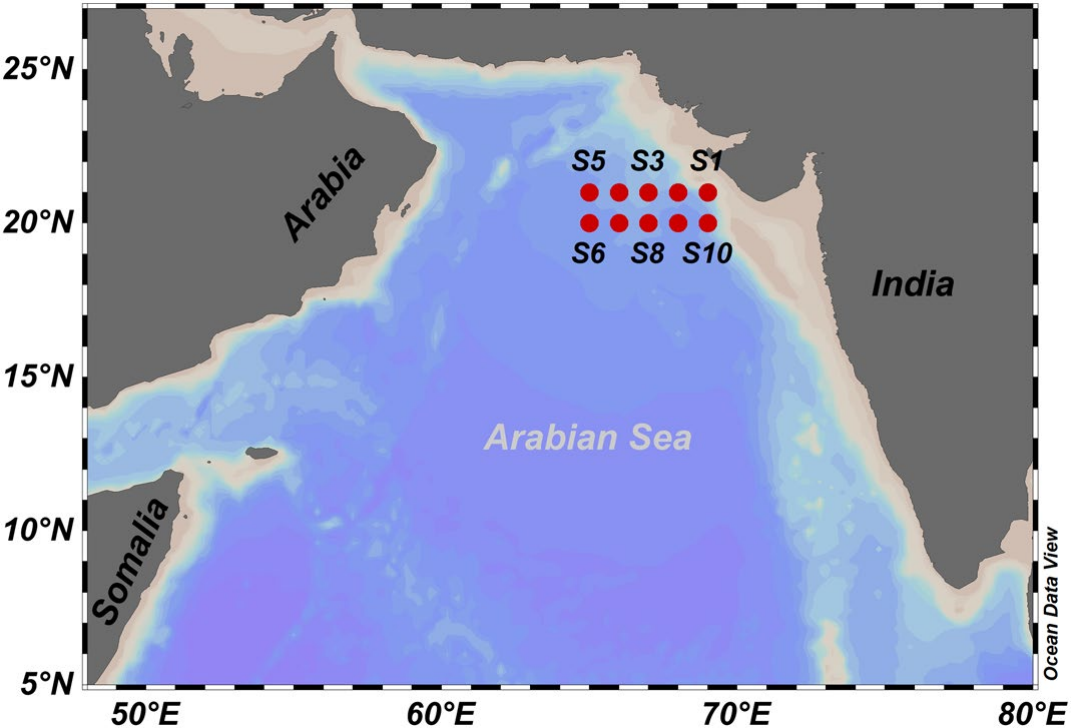
Map of the Arabian Sea showing sampling locations (S1 to S10). Map created in ODV^57^.

Rixen et al.^32^ documented that, deep convective mixing in the northern Arabian Sea during peak winter monsoon causes light limitation on the primary production by carrying photoautotrophic organisms below the euphotic zone. A similar condition is already reported in the Sargasso Sea, another region that experiences winter convection. The deeper mixed layer that ranges between 150 and 250 m in the Sargasso Sea restricts the primary production owing to the limited availability of photosynthetically active radiation throughout the mixed layer^33^. Nevertheless, some recent studies have contradicted the Sverdrup critical depth hypothesis showing an increase in the phytoplankton bloom during the mid-winter monsoon season using observational data^34–36^. Barber et al.^37^ and Marra and Barber^38^ using in situ data from the JGOFS expedition, argued that Sverdrup critical depth criterion does not limit primary production in the northern Arabian Sea during the winter monsoon. The present study was undertaken in the northern Arabian Sea during winter monsoon along 20°N and 21°N transects (Fig. 1) to describe the physical, chemical, optical, and biological water column properties and to understand the interplay of physics and biology on the initiation of a phytoplankton bloom.

## Results and discussions

**Figure 2:**
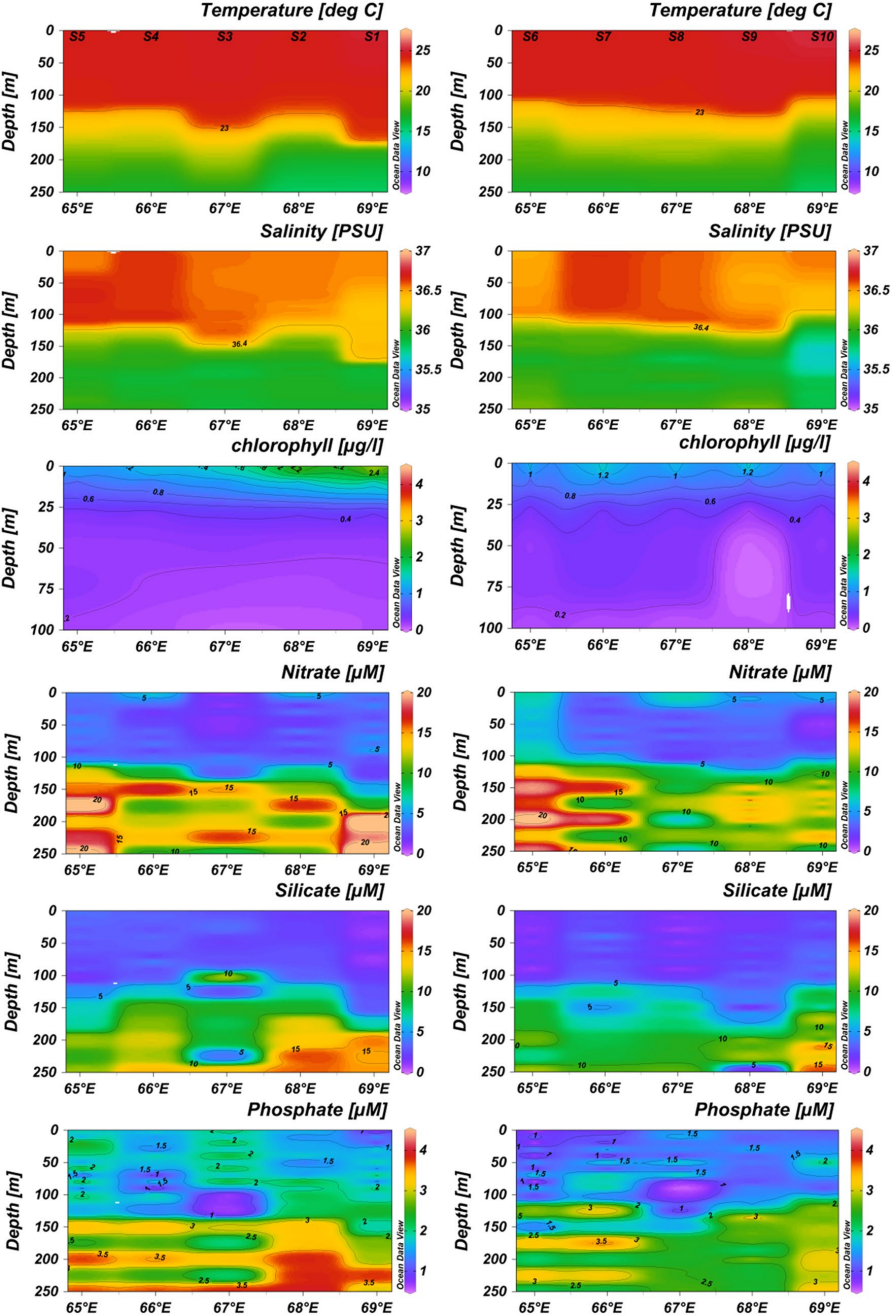
Vertical sections of Temperature (deg C), Salinity (PSU), chlorophyll (µg/l), Nitrate (µM), Silicate (µM) and Phosphate (µM) along 21°N (left panel) and 20°N (right panel) transects. Plots created using ODV^57^.

NAS experiences the widespread bloom of phytoplankton, during northern winter, owing to the conducive nutrient-rich environment. The spatial distribution of water temperature, and salinity observed along the two transects (21°N and 20°N) are shown in Fig. 2. The physical parameters showed cooler (SST ~ 24 °C), dense (salinity ~ 36.4), and well-mixed (MLD > 100 m) upper ocean.

The high saline water (~ 36.5–36.7 PSU), owing to the strong evaporation along with the cooler surface water, favours strong convective mixing leading to deepening of MLD^39^. The upper water column of the study area was well oxygenated at a saturation level of 95–103% (Supplementary Fig. 1). The thermocline (marked here as 23 °C isotherm) was a bit deeper along 21°N transect (~ 125 m) compared to 20°N transect (~ 110 m). These physical characteristics clearly indicate an active phase of convective mixing responsible for bringing nutrient-rich subsurface water to the upper layer^1,5,8^. The observed nitrate concentration in the upper layer during this time period was ~ 2.5 µM which was consistent with the earlier reports^24,25,40^ in NAS during the winter monsoon. The silicate concentration was also ~ 2.5 µM^2,25,40^ and phosphate concentrations varied between 1–2 µM (Fig. 2). The DIN concentration was, however, more than 5 µM.

According to Banse and Postel^41^, the winter convection is limited to 21º–23ºN, where the primary productivity is expected to be comparatively higher. However, the observational data indicate that, despite having a nutrient-enriched surface layer, the chl-*a* concentration was significantly lower (0.1–0.3 µg/l), in the upper water column, as compared to the earlier report^13^ (0.24–2.4 µg/l) (Fig. 2). The similar chl-*a* concentration (0.17 and 0.27 µg/l) was also reported by Bhattathiri et al.^42^ at stations J5 and J6, respectively, under the Indian-JGOFS programme during the winter monsoon^19^. Indian-JGOFS data also indicated diatom as the dominant phytoplankton group (~ 87%) during the winter monsoon. Summarizing all the data from extensive JGOFS field campaigns, by several countries in the Indian Ocean, Tarran et al.^43^ concluded that diatoms are the most dominant phytoplankton species in the Arabian Sea during the winter monsoon. However, few recent reports describe the dominance of *Noctiluca scintillans* in the northern Arabian Sea during the later phase of the winter monsoon^3,13,16,18,22^ which was not observed during JGOFS field campaigns. Recently Padmakumar et al.^24^ reported presence of microphytoplankton, both diatoms and *Noctiluca scintillans*, during the late winter monsoon in the open ocean waters of the northeastern Arabian Sea with a chlorophyll-*a* (chl-*a*) concentration of 1–2 mg m^−2^ and intensification of *Noctiluca* blooms during the early spring Inter monsoon.

**Figure 3:**
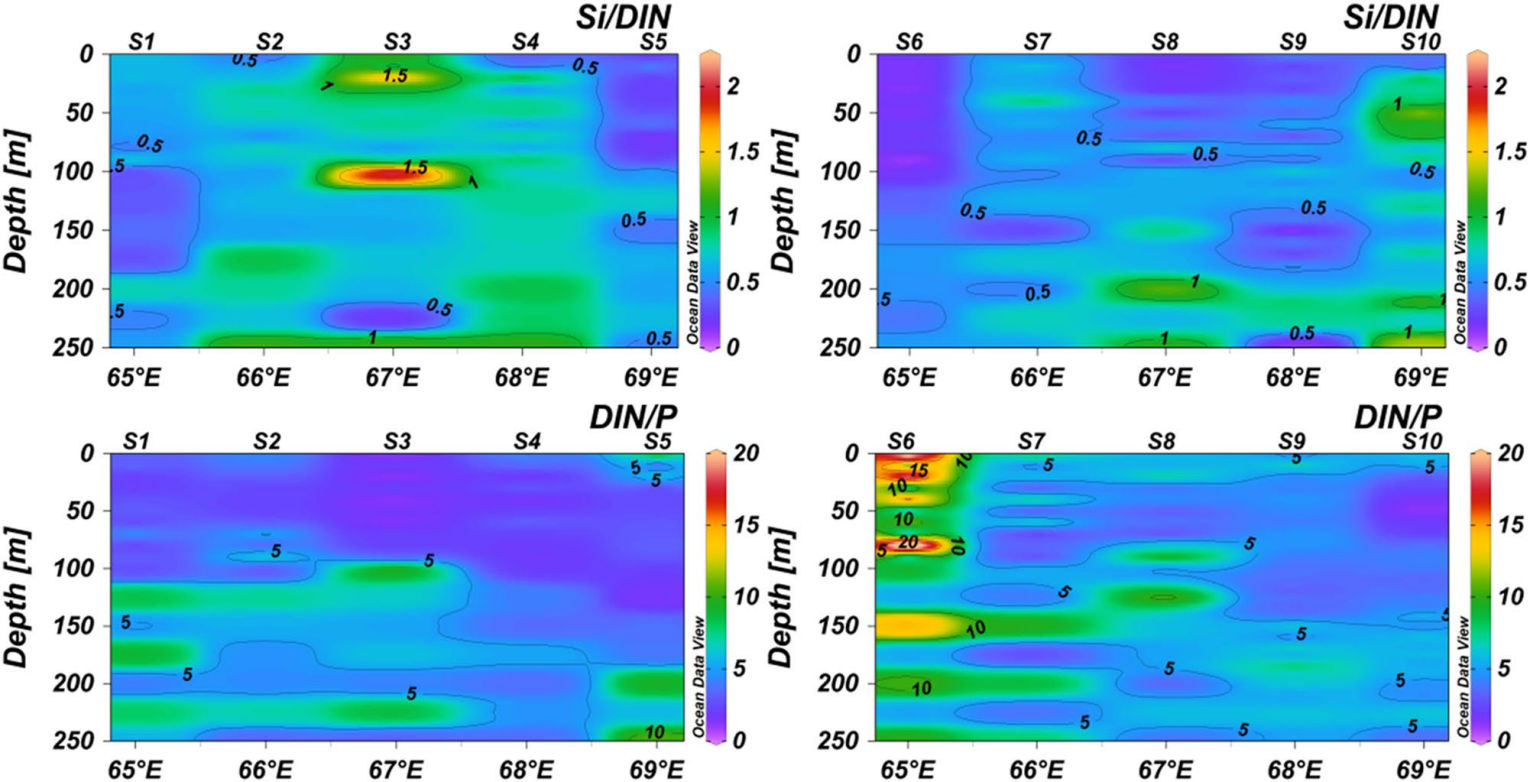
Vertical sections of Si/DIN and DIN/P along 21°N and 20°N transects. DIN includes nitrate, nitrite and ammonium. Plot created in ODV^57^.

Gomes et al.^18^ argued that massive outbreaks of *Noctiluca* bloom in the northern Arabian Sea is being facilitated by low oxygen waters. Subsequently, Prakash et al.^13^, showed, from both in-situ and Bio-Argo observations, that the surface layer of the northern Arabian Sea is well oxygenated during the winter monsoon. They further argued, using the climatological data that the skewed ratio (< 1) of silicate to nitrate inhibits proliferation of diatoms (since diatom frustules is composed primarily of silicate) and provides a niche for the proliferation of other species such as *Noctiluca*. Since the silicicline is deeper than the nitracline in the Arabian Sea, the strength of the convective mixing, through its manifestation on the mixed layer and consequent entrainment of nutrients in the upper mixed layer, defines the type of bloom likely to appear^3,13^. Since the hypothesis proposed by Prakash et al*.*^13^ was primarily based on climatological data having limited spatial and temporal coverage, we examined the silicate to DIN ratio from the data collected during this expedition. Our analysis of Si/DIN and DIN/P ratios along the two transects also show significant silicate limitation in the euphotic zone indicating “silicate-stressed” condition in NAS during the winter monsoon (Fig. 3). DIN/P ratio, however, did not show any phosphate limitation.

The proliferation of diatoms in such silicate-stressed conditions would be difficult and this will provide a niche for a bloom of other species such as dinoflagellates. Our observations also show a shallower nitracline (~ 110 m) compared to the silicicline (150–175 m) (Supplementary Fig. 2). The deeper MLDs are known to erode into the nutricline^37^ and entrain nutrients into the upper mixed layer. Since during the present expedition the MLD and nitracline were located at ~ 100–120 m, the MLD could penetrate into the nitracline. Since the silicicline was much deeper, MLD could not penetrate the silicicline^11,44,45^, the input of silicate was limited and therefore the Si/DIN ratio was skewed. This supports the hypothesis proposed by Prakash et al.^13^ that skewed Si/DIN ratio leads to the outbreak of *Noctiluca* bloom in this part of the world ocean replacing the diatom community. Though Vijayan et al.^40^ recently reported a strong bloom of diatoms in the northeastern Arabian Sea during the winter monsoon of 2018 but they encountered a favorable stoichiometric condition (Si/N =  ~ 1.5) for the proliferation of diatoms.

In general, the upwelled waters are with less concentration of silicate than nitrogen and the same has been reported by Gupta et al.^46^ in the Arabian Sea with Si:DIN < 0.7. In the present study, silicate concentrations are > 10 µM below 80 m where light is limiting. In the dim light region like at the base of the euphotic zone, mostly nano and picophytoplankton including some diatoms can grow with a special ability to photosynthesize under low light conditions. At 80 m depth, they are exposed to higher nitrogen than silicate. Importantly, nitrate uptake in marine phytoplankton is light-dependent, and therefore, under low light, they may possess a reduced nitrate uptake rate compared to the surface. Therefore, the silicate-stressed condition attributed to shoaling of MLD fuels phytoplankton (other than diatom) bloom holds true for the euphotic zone.

The observational data during the present expedition indicated favourable condition for *Noctiluca* bloom. However, no such signature was observed as reported earlier in the same geographical area^3,13,23^. On the contrary, the area was found to be dominated by picophytoplankton (77–85%). The HPLC resolved pigments suggested the prevalence of *Synechococcus*, one of the photosynthetic picocyanobacteria in the study region. A higher abundance of picophytoplankton with a predominance of *Synechococcus* has also been reported during the winter period in the northern Arabian Sea. Chndrasekhararao et al.^27^ also observed low microphytoplankton concentration in 2017 when the Si/DIN ratio was low and suggested that Si/DIN ratio controls the phytoplankton composition in the northeastern Arabian Sea. The absence of diatoms^19^ and *Noctiluca*^22^, which are known to contribute towards high productivity during the winter monsoon, had a bearing on the column productivity. The integrated chl-*a* concentration up to euphotic depth (Z_eu_: depth at which the irradiance reached 1% of the surface value) was very less (~ 40 mg/m^2^) despite having a very high nitrate concentration in the column (~ 200–300 mmol/m^2^) (Table 1).

**Table 1:** Station-wise distribution of integrated nitrate (NO_3_) concentration (mmol/m^2^), integrated chlorophyll-*a* (chl-*a*) concentration (mg/m^2^), surface silicate/dissolved inorganic nitrogen (Si/DIN) ratio, micro, nano and picophytoplankton biomass concentrations (µg/l).

Station no.	Date	Location	Integrated NO_3_	Integrated chl-*a*	Surface Si: DIN	Micro	Nano	Pico
S1	04–02-19	69E–21N	289.1	35.5	0.175	0.13	0.51	3.58
S2	05–02-19	68E–21N	248.7	30.3	0.355	0.22	0.61	3.27
S3	06–02-19	67E–21N	132.7	31.6	4.188	0.13	0.21	1.55
S4	07–02-19	66E–21N	253	27.4	0.426	0.09	0.25	1.74
S5	08–02-19	65E–21N	298	43.5	0.948	0.14	0.23	1.31
S6	09–02-19	65E–20N	494.6	66.1	0.305	0.15	0.27	1.38
S7	10–02-19	66E–20N	246.2	34.8	0.604	0.12	0.25	1.84
S8	11–02-19	67E–20N	338.7	43.1	0.306	0.13	0.29	1.51
S9	12–02-19	68E–20N	309.4	25.3	0.851	0.12	0.22	1.84
S10	13–02-19	69E–20N	207.6	43.8	0.552	0.16	0.22	1.39

**Figure 4:**
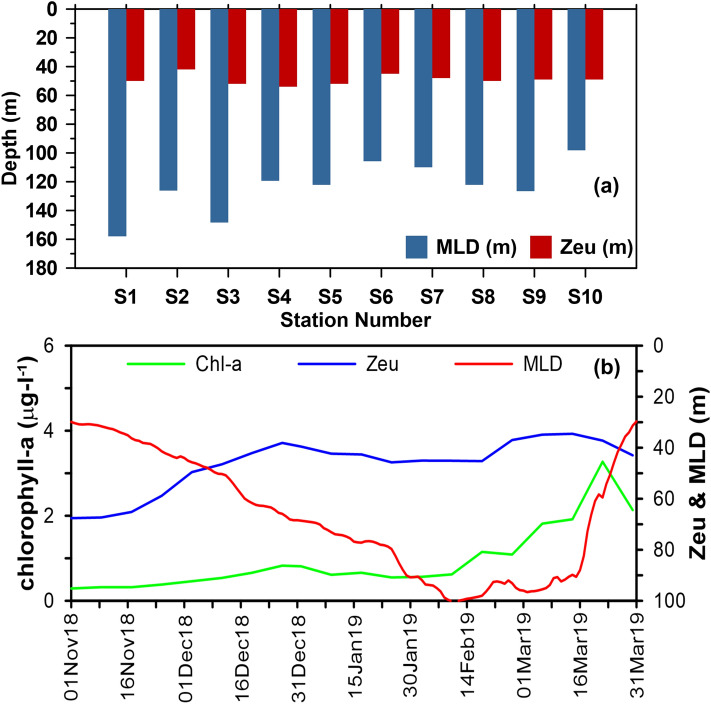
**(a)** Bar diagram of euphotic depth (red) and mixed layer depth (blue) at 10 sampling stations, **(b)** time-series of simulated mixed layer depth (INCOIS- GODAS), satellite derived euphotic depth and surface chl-*a*.

The central question still remained unanswered that why chl-*a* concentration is low despite high nutrient in the upper mixed layer? Apart from the nutrient, light also plays an important role in defining the water column production. The deep mixed layer does provide sufficient nutrients to fuel the productivity but is known to regulate the timing of the bloom, if the MLD is deeper than the Z_eu_, particularly in polar and temperate areas^28,30,47^. Various authors have evaluated the Sverdrup formulation of critical depth in context to the spring bloom in North Atlantic and other basins (e.g., review by Sathyendranath, Ji and Browman^48^). Though Behrenfeld et al.^34^ discarded the Sverdrup’s hypothesis in the subarctic Atlantic using 9 years of satellite observation and postulated an alternate ‘Dilution-Recoupling’ hypothesis for understanding the dynamics of the winter Atlantic bloom, he cautioned that the applicability of this new hypothesis needs to be tested for other basins. Barber et al*.*^37^ and Marra and Barber^38^, examined the interactions of the deep mixed layer, irradiance, nutrient transport, and productivity using the JGOFS synthesis data and concluded that Sverdrup’s critical depth limitation on productivity does not hold true in the Arabian Sea during the winter monsoon. Marra and Barber^38^ further emphasized that vertical mixing is never deeper than critical depth to limit phytoplankton growth. These evaluations were either based on the measured parameters such as specific growth rates^37^ or using numerical simulation with the help of published data on compensation irradiance from other basins^38^. We had also measured the sub-surface light field using a hyperspectral radiometer (Satlantic HyperPro-II) during the present expedition. Our analysis of the MLD and Z_eu_ shows that MLD was much deeper (~ 100–120 m) compared to the Z_eu_ (~ 42–54 m; mean = 49 ± 3.5 m; Fig. 4a).

Though the deeper MLD helps to bring lots of nutrients into the upper layer, the shallower Z_eu_ does not allow phytoplankton to use the nutrients efficiently as they will not get enough time in the upper sunlit layer. Since Z_eu_ also is the depth of critical irradiance, it can be considered as critical depth^49^. Our data shows that Sverdrup’s critical depth hypothesis holds true in the northern Arabian Sea during the winter monsoon. Deeper MLD, shallower Z_eu_, and skewed Si/DIN ratio explain the observed low chl-*a* in the NAS during the winter monsoon of 2019. We believe that for similar reasons, Bhattathiri et al.^42^ had reported low surface chl-*a* during winter monsoon expedition in the Arabian Sea experiencing similar conditions. The outbreak of massive blooms in this part of the world ocean is well documented, particularly in the satellite era. The question, however, still remains what causes such blooms? To understand the processes that govern the initiation of bloom in the northern Arabian Sea, we analysed MLD from the model (using INCOIS-GODAS simulations) and satellite (MODIS) estimated euphotic depth and surface chl-*a*. Figure 4b shows the time series of MLD, Z_eu_, and surface chl-*a* during November-March 2019. Though the Z_eu_ has not varied much (49.5 ± 13.5 m) during the winter monsoon of 2019, the MLD started deepening from < 40 m, during early November to ~ 100 m, during mid-February. The surface chl-*a* varied between 0.2 and 2 µg/l (0.83 ± 0.57 µg/l) showing low values during early November to mid-February and started increasing rapidly towards the latter half of February. The increasing surface chl-*a* was concurrent with the shallowing of the mixed layer. The bloom appears to have occurred towards the 3rd week of March 2019. In order to decipher whether it was specific to 2019 or this process occurs every year, we analysed the data for the 2016–2019 period and found that every year the increase in chl-*a* concentration was concurrent with the shallowing of the mixed layer. This clearly suggests that the initiation of bloom depends on the re-stratification of the water column. Though earlier workers have underplayed the role of Sverdrup’s critical depth hypothesis in the Arabian Sea^37,38^, they did emphasize on the importance of water column stability for optimal phytoplankton productivity.

**Figure 5:**
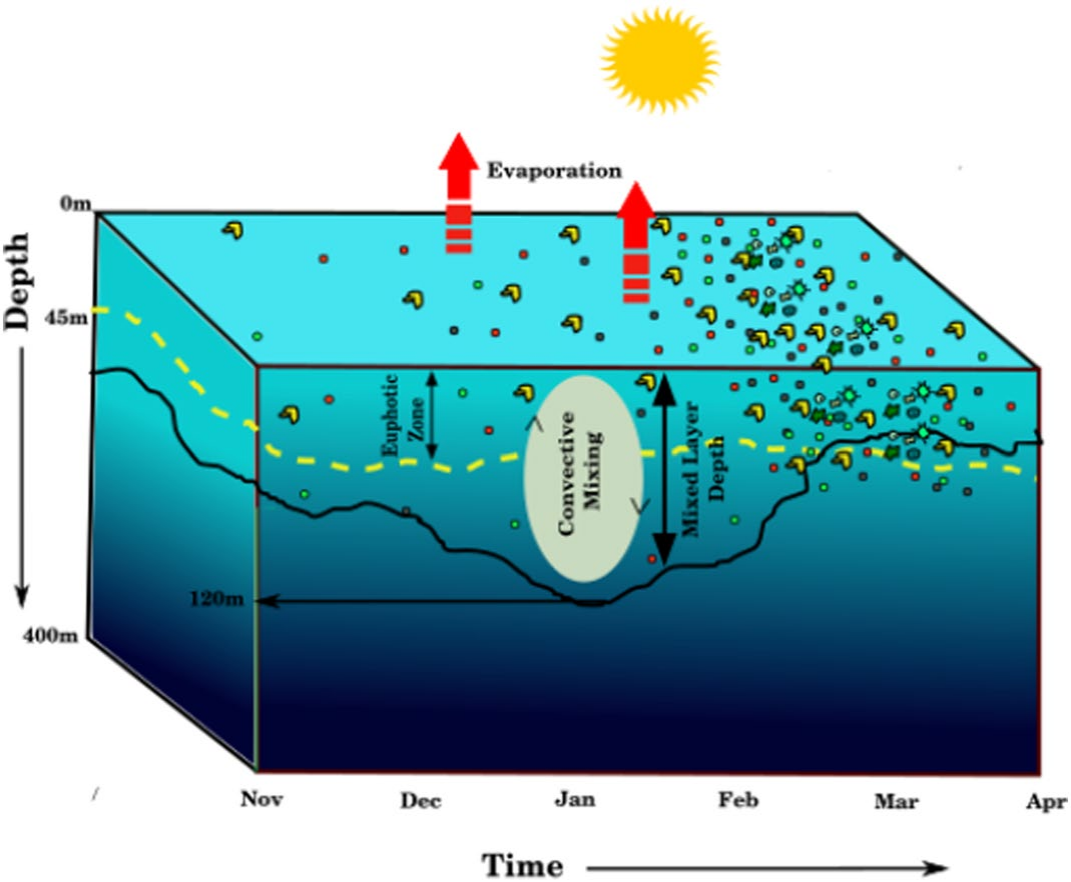
Schematic diagram which shows the interplay between mixed layer depth and euphotic depth on the initiation of phytoplankton blooms.

The significant outcome of the present study is that two factors primarily control the phytoplankton bloom in the northern Arabian Sea during the winter monsoon i.e. (1) the stoichiometric ratio of nutrients, mainly Si and DIN, input into the mixed layer, and (2) re-stratification of the euphotic zone during the later winter monsoon (Fig. 5). The dominance of heterotrophic dinoflagellate, *Noctiluca* is known to alter the food chain by feeding on diatoms as well as smaller zooplankton. This subsequently, has a large impact on the regional fisheries, apart from other factors such as chocking of gills, on the dissolved oxygen concentration^26,50^. The model simulated MLD and satellite-derived Z_eu_ can be very well used to predict the initiation of winter bloom in the Arabian Sea.

## Summary

The physical and biological interaction on the phytoplankton blooming was studied using in-situ data collected from the northeastern Arabian Sea during the winter monsoon of 2019. The observational data show a cooler, denser surface layer with a deep mixed layer and nutrient-rich upper surface layer. The upper water column was well-oxygenated with a saturation level of 95–103%. Despite harbouring higher nutrients, the chl-*a* concentration was lower than expected in the upper water column during the winter monsoon and was dominated by picophytoplankton, though the conditions were favourable for the *Noctiluca* to proliferate. Our analysis indicates a “silicate-stressed” condition with Si/DIN ratio of less than 1. The euphotic depth being shallower than the MLD suggests towards the Sverdrup’s critical depth limitation in the northeastern Arabian Sea during the winter monsoon. Despite having sufficient amount of nutrients, the chl-*a* concentration was low owing to Sverdrup’s critical depth limitation. Lower Si/DIN ratio and deeper MLD compared to Z_eu_ explains the observed low chl-*a* concentration in the study region. Our analyses using INCOIS—GODAS MLD, satellite-derived euphotic depth, and surface chl-*a* for the period 2016–2019 indicate that the phytoplankton bloom occurs towards the later phase of the winter monsoon when the MLD starts shallowing and becomes comparable with the euphotic depth. The blooming of phytoplankton in the NAS during the winter monsoon appears to be governed by re-stratification of the water column and the stoichiometric ratio (especially Si/DIN ratio) of nutrients in the euphotic zone during the late winter monsoon.

## Data and methods

### In situ observation

A scientific cruise, onboard FORV *Sagar Sampada* (cruise ID: SS383) was undertaken during the winter monsoon period (4th to 13th February 2019) in the NAS (Fig. 1). A Conductivity-Temperature-Depth (CTD; make: Sea-Bird Scientific, model: 19Plus) along with Fluorometer (make: Sea-Bird Scientific, model: ECO FLNTU) and optode (make: JFE Advantech Co. LTD., model: RINKO III) was used to measure the profiles of temperature, salinity, chl-*a* and dissolved oxygen (DO). The discrete water column sampling was carried out at 10 stations using a rosette sampling system fitted with Niskin bottles. Water samples were collected at six hours interval per day at each station up to 1000 m depth with the upper 100 m being sampled at an interval of 10 m. For estimation of chl-*a*, a known volume of water sample was filtered onto glass fiber filters (47 mm diameter with pore size 0.7 μm) and stored in liquid nitrogen until downstream analysis. The concentration of chl-*a,* from water samples, was analyzed using a High-Performance Liquid Chromatography (HPLC) system (make: Waters) as described in Srichandan et al.^51^. For size-fractionated chl-*a* concentration, a known volume of water sample were sequentially filtered using filters of different pore sizes (20 μm > 2 μm > 0.2 μm) to determine the contribution of chlorophyll-*a* concentration of three phytoplankton size classes (microphytoplankton (> 20 μm), nanophytoplankton (2–20 μm) and picophytoplankton (0.2–2 μm)). Subsequently, the filtrates were extracted using 90% acetone for 24 h, centrifuged at 2000 rpm and analyzed using a UV–Visible Spectrometer (make: Shimadzu, model: UV2600). The size-fractionated chl-*a* concentration was then calculated based on Strickland and Parsons^52^. The DO concentration, from water samples, was measured by adopting the Winkler titration method^53^. The titrimetric DO was validated with DO concentration obtained from oxygen optode sensor attached to CTD profiler. Macronutrients such as nitrate, nitrite, ammonium, phosphate, and silicate were analyzed colorimetrically using a UV–Visible Spectrometer^53^ (make: Shimadzu, model: UV2600). The downwelling irradiance (E_d_) and photosynthetically available radiance (PAR) were measured at each station using a hyperspectral optical profiler (make: Satlantic, model: HyperPro II). The Z_eu_ was calculated from PAR by considering the depth at which the PAR reaches 1% of its surface value. MLD was calculated as the depth at which the water temperature is 1ºC less than the surface temperature^9^.

### Satellite observation

The Z_eu_ was calculated using the diffuse attenuation coefficient of downward irradiance at 490 nm (K_d_(490)) with a spatial resolution of 4 km obtained from MODIS-Aqua (https://hermes.acri.fr/index.php?class=archive).

The euphotic depth was calculated using the exponential equation of light intensity,

1 $$ {\text{I }} = {\text{I}}_{{\text{o}}} {\text{exp }}\left( { - {\text{k}}_{{{\text{PAR}}}} {\text{Z}}} \right) $$

where ‘I’ is the intensity of light at depth ‘z’, ‘I_o_’ is the intensity at the surface and ‘k_PAR_’ is the diffuse attenuation coefficient of photosynthetically active radiation which is computed following Lotliker et al^54^.

2 $$ {\text{k}}_{{{\text{PAR}}}}  = {\text{ }}0.0{\text{168 }} + {\text{ }}0.{\text{97 k}}_{{\text{d}}} \left( {{\text{49}}0} \right) $$

where k_d_(490) is the downward irradiance at 490 nm. The Zeu, the depth at which the light intensity decreases to 1% of that at the surface, was calculated using the equation as follows.

3 $$ {\text{Z}}_{{{\text{eu}}}}  = {\text{ 4}}.{\text{61}}/{\text{k}}_{{{\text{PAR}}}} $$

The weekly composite of quality controlled surface chl-*a* concentration from MODIS-Aqua (https://hermes.acri.fr/index.php?class=archive) is also used in the present study. Further, temperature from INCOIS-GODAS (Indian National Centre for Ocean Information Services-Global Ocean Data Assimilation System) simulation^55,56^ is also used inorder to calculate the MLD.

### Data analysis and plots

Figures 1, 2, 3, 4 and 5 are plotted using Ocean Data View (ODV)^57^ software (version 5.0.). Figure 4(a) is made using MS Excel 2007 and Fig. 4(b) is made using Grapher 8. Figure 5 is made using Inkscape software (version 1.0.1; https://www.Inkscape.org).

## Supplementary Information

Supplementary Figures.

## Data Availability

The in situ data collected during the winter monsoon of 2019 is available from Indian National Centre for Ocean information Services (INCOIS) on request. The euphotic Depth and chl-*a* concentration are downloaded from MODIS-AQUA (https://hermes.acri.fr/index.php?class=archive). INCOIS-GODAS simulation used for MLD estimation is available at INCOIS Live Access Server (las.incois.gov.in).
