# Supplementary material for: Physicochemical controls on the initiation of phytoplankton bloom during the winter monsoon in the Arabian Sea
Source: Sci Rep. 2021 Jun 29;11:13448. doi: 10.1038/s41598-021-92897-3 (PMC8242075; doi:10.1038/s41598-021-92897-3)

**Supporting Information for**

“**Physicochemical controls on the initiation of phytoplankton bloom during the winter monsoon in the Arabian Sea**”

^1,2^R. S. Lakshmi, ^1*^Satya Prakash, ^1^Aneesh A. Lotliker, ^1^Sanjiba K. Baliarsingh, ^1^Alakes Samanta, ^1,2^Teesha Mathew, ^1^Abhisek Chatterjee, ^3^Biraja K. Sahu, ^1^T. M. Balakrishnan Nair

^1^Indian National Centre for Ocean Information Services, Ministry of Earth Sciences, Government of India, Hyderbad-500090, India

^2^Kerala University of Fisheries and Ocean Studies (KUFOS), Kochi-682506, India

^3^Atal Centre for Ocean Science & Technology for Islands, National Institute of Ocean Technology, Ministry of Earth Sciences, Government of India, Port Blair-744103, Andaman and Nicobar Islands, India

*Email of corresponding author: [satyap@incois.gov.in](mailto:satyap@incois.gov.in)

**Figure S1.** Vertical section of Dissolved Oxygen (μmol/kg) and Oxygen Saturation (%) along 21°N and 20°N transects.


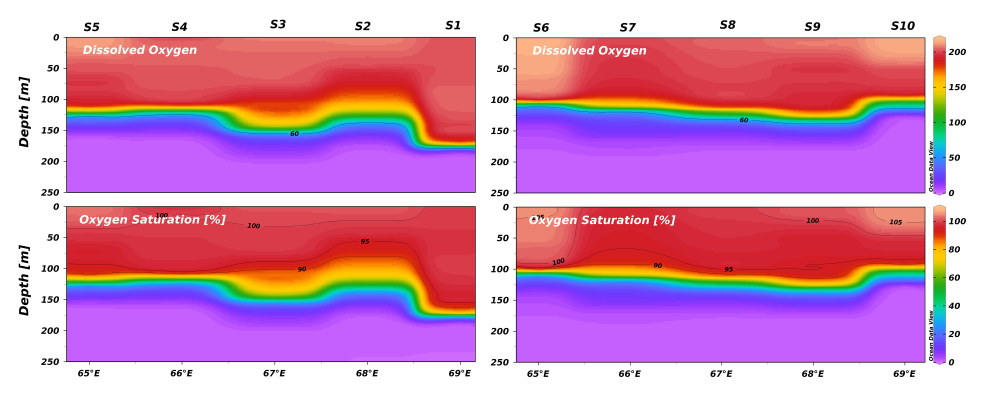


**Figure S2**. Profile of Nitrate (Blue) and Silicate (Red) in μM at Station S5.


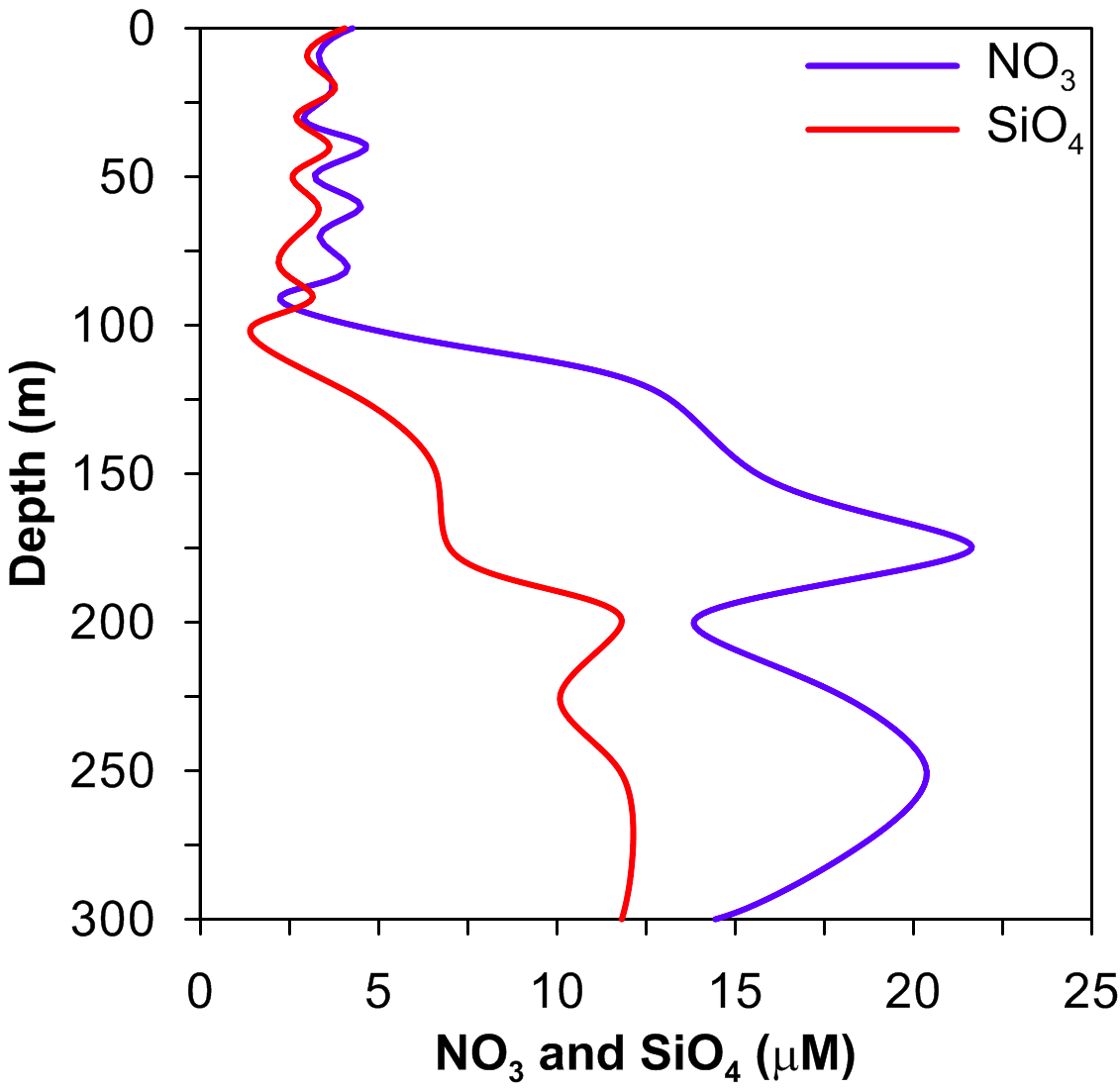

Supplement: Supplementary file 1 — Supplementary Figures. [file 41598_2021_92897_MOESM1_ESM.docx]
